# IDRdecoder: a machine learning approach for rational drug discovery toward intrinsically disordered regions

**DOI:** 10.3389/fbinf.2025.1627836

**Published:** 2025-07-18

**Authors:** Clara Shionyu-Mitusyama, Satoshi Ohmori, Subaru Hirata, Hirokazu Ishida, Tsuyoshi Shirai

**Affiliations:** ^1^ Department of Bioscience, Nagahama Institute of Bio-Science and Technology, Nagahama, Shiga, Japan; ^2^ Faculty of Data Science, Shiga University 1-1-1 Banba, Hikone, Shiga, Japan

**Keywords:** intrinsically disordered proteins, neural net, sequence-based prediction method, structural bioinformatics, drug design

## Abstract

**Introduction:**

Intrinsically disordered regions (IDRs) of proteins have traditionally been overlooked as drug targets. However, with growing recognition of their crucial role in biological activity and their involvement in various diseases, IDRs have emerged as promising targets for drug discovery. Despite this potential, rational methodologies for IDR-targeted drug discovery remain underdeveloped, primarily due to a lack of reference experimental data.

**Methods:**

This study explores a machine learning approach to predict IDR functions, drug interaction sites, and interacting molecular substructures within IDR sequences. To address the data gap, stepwise transfer learning was employed. IDRdecoder sequentially generate predictions for IDR classification, interaction sites, and interacting ligand substructures. In the first step, the neural net was trained as autoencoder by using 26,480,862 predicted IDR sequences. Then it was trained against 57,692 ligand-binding PDB sequences with higher IDR tendency via transfer learning for predict ligand interacting sites and ligand types.

**Results:**

IDRdecoder was evaluated against 9 IDR sequences, which were experimentally detailed as drug targets. In the encoding space, specific GO terms related to the hypothesized functions of the evaluation IDR sequences were highly enriched. The model’s prediction performance for drug interacting sites and ligand types demonstrated the area under the curve (AUC) of 0.616 and 0.702, respectively. The performance was compared with existing methods including ProteinBERT, and IDRdecoder demonstrated moderately improved performance.

**Discussion:**

IDRdecoder is the first application for predicting drug interaction sites and ligands in IDR sequences. Analysis of the prediction results revealed characteristics beneficial for IDR-drug design; for instance, Tyr and Ala are preferred target sites, while flexible substructures, such as alkyl groups, are favored in ligand molecules.

## 1 Introduction

The continuous decline in productivity in the research and development of novel pharmaceutical drugs has been noted (Pammolli et al., 2011; Williams, 2011). One complex cause of this decline is believed to be the depletion of easily accessible drug targets, often referred to as low-hanging fruits. These circumstances have driven the demand for new drug targets or modalities (Kiriiri et al., 2020). “Drugging the undruggable proteins” is a central challenge in efforts to identify new protein targets that were previously avoided or overlooked. A prominent group among these neglected targets is intrinsically disordered proteins (IDPs) or regions (IDRs) (Biesaga et al., 2021; Hassin and Oren, 2023).

IDRs are protein regions that lack a defined structure under native conditions. Although the biological significance of these unstructured proteins took time to be recognized, IDRs are now understood to play crucial roles in several biological processes, primarily in molecular recognition, signal transduction, and liquid–liquid phase separation in cells (Tompa, 2011; Oldfield and Dunker, 2014). Notably, many mutations in IDRs are pathogenic (Uversky et al., 2008; Darling and Uversky, 2017; Shigemitsu and Hiroaki, 2018). Approximately 35% of the human proteome comprises IDRs, and 22%–29% of disease-associated missense mutations occur within these regions (Vacic et al., 2012; Hijikata et al., 2017).

These developments have driven research in IDR-targeted drug discovery (Ruan et al., 2019; Santofimia-Castano et al., 2020; Saurabh et al., 2023). To date, pioneering studies in this field have focused on targets such as amyloid beta (Aβ) (Scherzer-Attali et al., 2010; Convertino et al., 2011), androgen receptor (AR) (Sadar, 2020), PTP1B (Krishnan et al., 2014), TipA (Habazettl et al., 2014), alpha-synuclein (αSyn) (Toth et al., 2014; Tatenhorst et al., 2016), cMyc (Follis et al., 2008; Yu et al., 2016), p27 (Iconaru et al., 2015), NUPR1 (Neira et al., 2017), and p53 (Ruan et al., 2020). In most of these studies, drug candidates were identified through experimental screenings aimed at inhibiting functions and/or protein interactions. A few studies—specifically those targeting Aβ (Convertino et al., 2011), p53 (Ruan et al., 2020), αSyn (Toth et al., 2014), cMyc (Yu et al., 2016), and NUPR1 (Neira et al., 2017) also incorporated rational approaches. Typically, these rational methods combine conformational searches of IDR sequences using molecular dynamics simulations with ligand searches through fragment-based docking simulations. However, none of the potential drugs identified in these studies have received approval for their intended use.

Various computational techniques have been developed to predict, classify, and identify interaction sites within IDRs. These functional regions are often termed molecular recognition fragments (MoRFs) or short linear sequence motifs (SLiMs). MoRFs and SLiMs play essential roles in binding to proteins, nucleic acids, and lipids (membranes). While these elements are primarily studied for their specific interactions with intrinsic native macromolecules, their potential roles in binding extrinsic small molecules, such as pharmaceutical drugs, are frequently overlooked. This oversight may stem from the limited experimental data currently available on IDR-drug interactions. Moreover, existing computational methods for drug discovery and design predominantly follow the “lock-and-key” model, which is better suited for structured proteins.

In recent years, neural network-based machine learning, including transformers, has become the mainstream, and is achieving significant results, for IDR classification and interacting site prediction (Chen et al., 2022; Basu et al., 2023). However, the lack of training data still remains a major bottleneck in IDR-targeted drug design. Therefore, rational methods for IDR-drug discovery and design are critically lacking, and advancing such approaches would significantly benefit the field of drug development against novel target proteins. In this report, a preliminary method, named IDRdecoder, was developed to predict drug interaction sites and potential interacting ligands on IDR sequences using a neural network-based machine learning approach. This method was designed to address and compensate for the existing data gap in IDR-drug interactions by transfer learning and stepwise predictions of IDR classification, drug interacting sites, and ligand types.

## 2 Methods

### 2.1 Data sets

The IDR amino acid sequences were obtained from the RefSeq (GCF) genome assembly database (O'Leary et al., 2016). The translated ORF sequences were analyzed using IUPred2A, and a sequence region was classified as an IDR if at least 30 consecutive residues had a score exceeding 0.9 (Meszaros et al., 2018). In total, 26,480,862 sequences with an average length of 109 residues were extracted from the proteomes of 23,041 species, forming a dataset referred to as DS-IDR (Supplementary Figure S1a; Supplementary Table S1).

The data for drug-interacting sites of IDRs and their corresponding potential drug formulas were collected from literature documenting drug discovery efforts targeting amyloid beta (Aβ) (Scherzer-Attali et al., 2010), androgen receptor (AR) (Sadar, 2020), PTP1B (Krishnan et al., 2014), TipA (Habazettl et al., 2014), alpha-synuclein (αSyn) (Tatenhorst et al., 2016), cMyc (Follis et al., 2008), p27 (Iconaru et al., 2015), NUPR1 (Neira et al., 2017), and p53 (Ruan et al., 2020). In total, nine sequences (averaging 72 residues) with 130 interacting sites and 11 chemical formulas of potential drugs were obtained (Supplementary Table S1; Supplementary Figure S2). These data were used to create the primary validation dataset, referred to as DS-IDR-V. MarvinSketch 20.19 (2020) by ChemAxon (http://www.chemaxon.com) was used to construct the coordinates of the chemical structures.

The sequences of protein segments interacting with ligands were extracted from the PDB as of 21 January 2021 (wwPDB Consortium, 2019). Complete subunit sequences were randomly divided into segments to ensure that their length distribution matched that of disordered sequences in DS-IDR (Supplementary Figure S1b). The IDR tendencies of these segments were evaluated using IUPred2A (Meszaros et al., 2018). For each segment, the ligand-interacting sites, ligand identity, and chemical formulas were recorded. These chemical formulas were further divided into protogroups, defined as small chemical compounds in the PDB that could act as standalone protein ligands (e.g., benzene, butanol) and appear frequently as substructures within larger ligands. Chemical formulas for 32,414 protein-ligand molecules were curated from the PDB. The frequency with which each ligand matched parts of other ligands was determined using a graph match algorithm (Saito et al., 2012). Ligand molecules (potential protogroups) were ranked according to their match frequency (Supplementary Figures S1e, S3). Initially, ligands composed of five or more atoms were preferentially selected, resulting in 61 protogroups that covered 71.2% of all PDB ligand atoms, with a 34.6% overlap (fraction of atoms assigned to more than two protogroups). After lowering the threshold to four or more atoms, an additional 26 protogroups were selected, increasing coverage to 78.1% with a 50.5% overlap. In total, 87 protogroups (comprising four or more atoms) were used as prediction targets (Supplementary Table S5).

Amino acid residues with at least one atom within 4.0 Å of a protogroup atom were defined as interacting sites. A total of 961,840 sequences (averaging 112 residues) with 3,002,920 interacting sites for 87 protogroups were extracted (Supplementary Table S1). This dataset, referred to as DS-PDB, was further divided into sub-datasets. The training dataset (DS-PDB-T) consisted of 57,448 sequences showing a relatively higher IDR tendency. These sequences either had an IUPred2A score above 0.5 or were randomly selected with decreasing probability for lower scores (as shown in Supplementary Figure S1c), resulting in 171,007 interacting sites (Meszaros et al., 2018). For additional validation, sequences not included in DS-IDR-V or DS-PDB-T were selected if their corresponding segments had structural evidence of disorder. This criterion required that the region was unmodeled in at least one experimental structure in the PDB, producing a dataset of 70 sequences with 259 interacting sites (DS-PDB-V, Supplementary Table S1). These segments were identified by comparing DS-PDB with disorder-annotated PDB sequence clustering data (Lobanov et al., 2020). Additionally, sequences not included in DS-PDB-T, DS-IDR-V, or DS-PDB-V and displaying a lower IDR tendency (IUPred2A scores ranging from 0.0 to 0.3) were randomly selected to create a negative dataset, referred to as DS-PDB-N. This dataset comprised 5,000 sequences (average 129 residues) with 18,060 interacting sites (Supplementary Table S1) (Meszaros et al., 2018).

### 2.2 Design and construction of machine learning model

**FIGURE 1:**
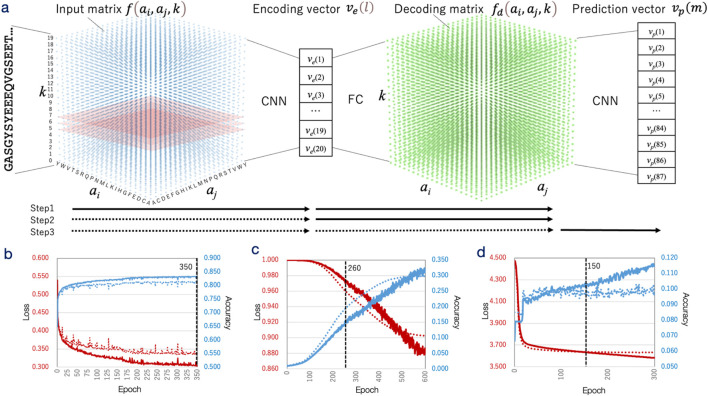
Architecture and training process of IDRdecoder. **(a)** Schematic representation of IDRdecoder. The input sequence is transformed into a matrix $f\left(a_{i},a_{j},k\right)$ and processed through convolutional layers (CNN) to generate an encoding vector $v_{e}\left(l\right)$ *,* classifying the input sequence (encoding phase, training step 1). This encoding vector connects to the decoding matrix $f_{d}\left(a_{i},a_{j},k\right)$ via dense layers (FC) for interaction site prediction (decoding phase, training step 2). The decoding matrix is further processed through CNN layers to produce the prediction vector $v_{p}\left(m\right)$ for interacting protogroup prediction (prediction phase, training step 3). The training is conducted in three distinct steps, with solid arrows indicating trained components and dotted arrows indicating fixed components during each step. **(b)** Learning curve of training step 1. The loss value (left axis, red) and accuracy (right axis, blue) are plotted against epochs up to 350. The dotted line marks the epoch where training stops (350). **(c)** Learning curve of step 2. **(d)** Learning curve of step 3.

The machine learning model developed in this study was designed to process IDR sequences as input and predict drug (small molecule) interacting sites on these sequences, along with the likely interacting protogroups, as output (Figure 1a). A straightforward neural network architecture was implemented in Python (ver. 3.10) using TensorFlow (ver. 2.12) library and was named IDRdecoder (Van Rossum and Drake, 2009; Abadi et al., 2016).

In the input layer, protein sequences of varying lengths were transformed into a three-dimensional (20 × 20 × 20) matrix

$$f\left(a_{i},a_{j},k\right)=n\left(a_{i},a_{j},k\right)/\sum_{i,j,k}n\left(a_{i},a_{j},k\right)$$

where $n\left(a_{i},a_{j},k\right)$ the number of residue pairs between specific amino acids $a_{i}$ and $a_{j}$ separated by k=j−i residues in the IDR sequence. Since the layer $n\left(a_{i},a_{j},0\right)$ was formed as a diagonal matrix reflecting amino acid frequency, $f\left(a_{i},a_{j},0\right)$ the matrix was divided by 20 to attenuate the values. Additionally, $f\left(a_{i},a_{j},k\right)$ was smoothed as

$$f\left(a_{i},a_{j},k\right)=\sum_{k-3\leq l\leq k+3}f\left(a_{i},a_{j},l\right)/4^{\left|k-l\right|}$$

The input matrix was processed through a single convolutional layer containing 800 filters with dimensions of 20 × 20 × 3 and a stride of 20 × 20 × 1. This convolutional layer was connected to two fully connected (dense) layers with dimensions of 400 × 1 and 20 × 1, respectively. The second dense layer produced the encoding vector $v_{e}\left(l\right)$ of the input sequence, forming the encoding part of the network, which spans from the input layer to the encoding layer. The encoding layer was further connected to two dense layers with dimensions of 75 × 1 and 1,200 × 1, leading to the decoding matrix $f_{d}\left(a_{i},a_{j},k\right)$ with dimensions of 20 × 20 × 20. This matrix provided predicted values for the interacting sites. This segment of the network, from the encoding layer to the decoding matrix, was defined as the decoding part.

The decoding matrix was subsequently passed through a single convolutional layer (mirroring the structure of the input convolutional layer), followed by a dense layer (400 × 1), and finally connected to the output layer (87 × 1). This output layer produced a prediction $v_{p}\left(m\right)$ vector corresponding to 87 protogroups, completing the prediction part of the network, which spans from the decoding layer to the output layer. For activation functions, the ReLU (rectified linear unit) function was applied throughout the network, except before the encoding layer, where the tanh (hyperbolic tangent) function was used to reduce extreme values, and before the output layer, where the softmax was employed.

In the first step, IDRdecoder was trained as an autoencoder (Hinton and Salakhutdinov, 2006; Yang et al., 2018; Tian et al., 2021) using the DS-IDR dataset. In this configuration, the input data at the input layer and the target data at the decoding layer were identical. The predicted IDR sequences were processed into input data as described above, and the encoding and decoding components (containing 17,852,080 weights) were trained using the Adam (adaptive moment estimation) optimizer. The Pearson correlation coefficient and 1 – |Pearson correlation coefficient| were used as the metrics and the loss function, respectively. Half of DS-IDR was randomly assigned to the test set. The learning rate was fixed to 1 × 10^−3^ during all epochs.

In the second step, IDRdecoder was trained for interacting site prediction using transfer learning. During this phase, 7,689,220 weights in the encoding layer were fixed with the values obtained from the first step, while 10,162,860 weights in the decoding layer were retrained. The dataset was switched to DS-PDB-T, with input data prepared from amino acid sequences as previously described. However, the target data in this step were designed to represent only the ligand-interacting sites. The learning rate was reduced to 7 × 10^−5^, while all other conditions remained the same as in the first step.

In the third step, IDRdecoder was trained to predict interacting protogroups. Here, all 17,852,080 weights in the encoding and decoding layers were fixed as obtained from the second step, and 7,750,573 weights in the prediction layer were trained. The DS-PDB-T dataset was again used, and the input data remained consistent with the second step. The target data were formatted as one-hot vectors, where the element corresponding to the interacting protogroup was set to 1. Categorical accuracy and categorical cross-entropy were employed as the evaluation metric and loss function, respectively. The adamax optimizer was used, with the learning rate further reduced to 1 × 10^−7^. The other conditions were the same as the second step. Additionally, the last step was also repeated by training all 25,602,653 weights for a comparison purpose.

### 2.3 Validation of machine learning model

IDRdecoder was evaluated across three aspects. First, the encoding vector $v_{e}\left(l\right)$ was expected to represent the classification of IDR sequences, with vector proximity indicating functional similarity among IDRs. To assess this, GO enrichment was examined for several IDRs with proposed functional roles. Seven IDR sequences were selected as queries: those of AR^*^ (Chen et al., 2023), αSyn (Hawk et al., 2019; Makasewicz et al., 2024), Rho (transcription termination factor) (Krypotou et al., 2023), SPT16 (FACT component) (Mayanagi et al., 2019), MDP1 (DNA-binding protein HupB) (Nishiyama et al., 2024), Tau (Connolly et al., 1977; Elie et al., 2015; Trushina et al., 2019), and Aβ^*^ (Ramaker et al., 2013; Tsoi et al., 2023). Although the proteins AR and Aβ were included in the validation set (DS-IDR-V), the IDR segments used for this analysis differed from those in DS-IDR-V (Supplementary Tables S1, S3).

The top 2,000 IDR sequences closest to each query in the encoding space were extracted from DS-IDR based on the Euclidean distance between encoding vectors $v_{e}\left(l\right)$ . IDR sequences of close homologs, identifiable via BLAST (Altschul et al., 1990) with an E-value below 1, were excluded. In the BLAST search, the query sequences were IDRs, and the database consisted of sequences from DS-IDR. Redundant sequences were removed by cross-referencing gene IDs. GO terms (Sayers et al., 2022; Gene Ontology et al., 2023) were assigned to the sequences using Gene2Refseq and Genes2Go (O'Leary et al., 2016). GO enrichment was evaluated using the P-value from a one-sided (greater) binomial test, comparing the occurrence rate of a GO term around a specific IDR to its rate across all examined queries. No threshold or compensation was applied for P-values. Each 20 GO terms having the lowest P-values were shown in Supplementary Table S2.

Second, the prediction performance of the decoding matrix $f_{d}\left(a_{i},a_{j},k\right)$ for interaction sites was evaluated. The matrix $f_{d}\left(a_{i},a_{j},k\right)$ was converted into a score for each residue *i* in the input sequence as

$$s\left(i\right)=\sum_{l\leq i,i-l\leq 20}f_{d}\left(a_{l},a_{i},i-l\right)+\sum_{l>i,l-i\leq 20}f_{d}\left(a_{i},a_{l},l-i\right)$$

where $a_{i}$ represents the amino acid at the input sequence. This score was further normalized to

$$s_{n}\left(i\right)=s\left(i\right)/\max\left\{s\left(l\right)\right\}$$

Ligand-interacting sites were predicted for the IDR sequences in DS-IDR-V. The predictions were also performed using IUPred2A (Meszaros et al., 2018), MoRF_CHiBi_ (Malhis and Gsponer, 2015), DeepDISOBind (Zhang et al., 2022), and ProteinBERT (Brandes et al., 2022), with their performances compared to that of IDRdecoder. For ProteinBERT predictions, the pre-trained model (downloaded from GitHub (https://github.com/nadavbra/protein_bert) was fine-tuned for the site prediction task using DS-PDB-T. Additionally, IDRdecoder was applied to predict interacting sites in DS-PDB-V and DS-PDB-N, and the performance across these datasets was compared.

Third, the predictive ability of the prediction vector $v_{p}\left(m\right)$ for interacting protogroups was assessed using DS-IDR-V. The prediction vector $v_{p}\left(m\right)$ was normalized for each sequence to yield a score $t_{n}\left(i\right)$ for protogroup *i* as

$$t_{n}\left(i\right)=v_{p}\left(i\right)/\max\left\{v_{p}\left(m\right)\right\}$$

This performance was compared with that ProteinBERT, fine-tuned for the category prediction task using DS-PDB-T. Similar to the interaction site prediction, IDRdecoder was also used to predict interacting protogroups in DS-PDB-V and DS-PDB-N, and the capabilities were compared.

Additionally, the true-positive rates (TPR) of IDRdecoder for each amino acid and protogroup were compared with IDR propensities. TPRs were calculated as the sum of true positives and false negatives across examined thresholds. IDR propensity represented the relative likelihood of participating in IDR interactions. For amino acids, propensity was defined as the fraction of true cases among the total occurrences of each amino acid in DS-IDR-V and DS-PDB-V, divided by the general ligand interaction propensity evaluated for ordered proteins (Soga et al., 2007). For protogroups, it was calculated as the fraction of true cases among the total occurrences of each protogroup in DS-IDR-V and DS-PDB-V, divided by the protogroup’s frequency in DS-PDB-T. These IDR propensities were scaled between 0.0 and 1.0, as shown in Figure 4.

All statistical analyses were performed using R (ver. 3.6.3) or Python with the SciPy library (ver. 1.10.1). (Team, 2007; Virtanen et al., 2020).

## 3 Results

### 3.1 IDR classification via encoding layer

A simple neural network, IDRdecoder, was developed to predict ligand interaction sites and ligand types directly from the amino acid sequences of IDR. IDRdecoder consists of three main components: encoding, decoding, and predicting modules. It is designed to sequentially generate predictions for IDR classification, interaction sites, and interacting ligand substructures (Figure 1a). To accommodate IDR sequences of varying lengths, the model converts sequences into a 3D matrix that captures the relative frequencies of amino acid pairs and their separation within the sequence. Initially, the encoding and decoding components were trained as an autoencoder using 26,480,862 predicted IDR sequences derived from 23,041 genomes (Figure 1b; Supplementary Figure S1a). Training continued until saturation by measuring the correlation coefficient between the input and decoded metrics.

**FIGURE 2:**
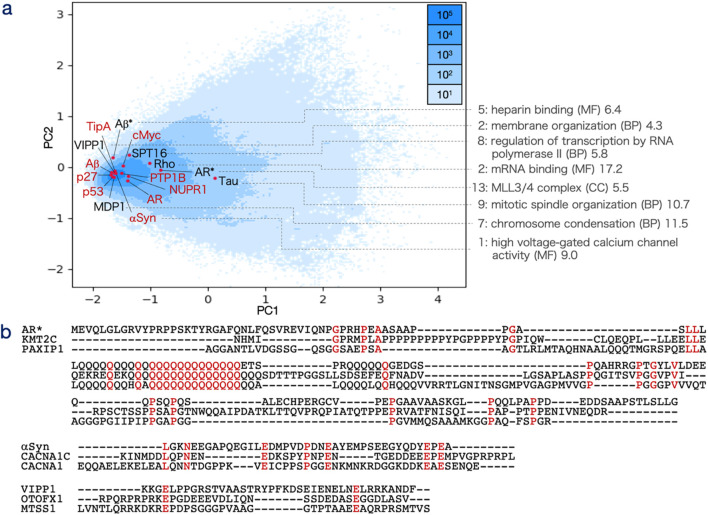
Principal component analysis (PCA) of encoding vectors. **(a)** Distribution of 26,480,862 IDR sequence encoding vectors plotted on the PC1 (horizontal axis) and PC2 (vertical axis) plane. The axis scales represent principal component loadings, with sequence density visualized by a blue color gradient. Red dots and labels indicate IDRs from the validation dataset (DS-IDR-V), while black dots and labels represent IDRs used in GO enrichment analysis. For these sequences, the most relevant enriched term is presented as rank: term (category) along with the -log_10_P-value. **(b)** Sequence alignments of representative IDRs: Top: Alignment of IDRs near AR* (Refseq ID: NP_000035.2, residues 343–448), associated with the “MLL3/4 complex (CC).” Aligned sequences include KMT2C (XP_043790783.1, residues 2,288–2,477, encoding vector distance: 0.464) and PAXIP1 (XP_040158493.1, residues 1,397–1,561, distance: 0.489). Consensus residues are highlighted in red. Middle: Alignment of IDRs near αSyn (NP_000336.1, residues 100–140), linked to “high voltage-gated calcium channel activity.” Aligned sequences are CACNA1C (XP_044917098.1, residues 850–892, distance: 0.125) and CACNA1 (XP_047501356.1, residues 694–747, distance: 0.127). Bottom: Alignment of IDRs near VIPP1 (NP_001322349.1, residues 221–259), associated with “membrane organization.” Aligned sequences include OTOFX1 (XP_042838266.1, residues 649–685, distance: 0.094) and MTSS1 (XP_024596750.1, residues 429–471, distance: 0.100).

The encoding module transformed IDR sequences into 20-dimensional real-valued encoding vectors. Upon completion of training, the correlation coefficients between the input and decoded matrices averaged 0.66 (with a standard error of 1.30 × 10^−3^) and had a standard deviation of 0.12, indicating that the encoding vectors effectively captured and reconstructed the input data (Supplementary Figure S1d). Principal component analysis (PCA) was applied to the encoding vectors, and the IDRs were visualized on the PC1–PC2 plane (Figure 2).

To assess whether the encoding vectors could classify IDRs based on function, several experimentally characterized IDR sequences specifically from AR*, αSyn, Rho, SPT16, MDP1 (DNA-binding protein HupB), Tau, and Aβ* were analyzed. These IDRs were generally distributed in alignment with the overall frequency distribution of all IDRs on the PC1–PC2 plane (Figure 2). To further explore functional similarities, non-homologous IDRs located near these reference IDRs (based on inter-vector distances) were extracted, and gene ontology (GO) enrichment analysis was performed (Supplementary Table S2).

Overall, the highly enriched GO terms varied among the IDRs, with specific terms related to the hypothesized functions of each IDR appearing among the significantly enriched categories. For example, “MLL3/4 complex (CC)” was enriched for AR, supporting its proposed role in enhancer complex assembly (Panigrahi and O'Malley, 2021; Chen et al., 2023). Similarly, “high voltage-gated calcium channel activity” was enriched for αSyn, aligning with the hypothesis that αSyn aggregation is modulated by calcium channels (Leandrou et al., 2019). For MDP1, “chromosome condensation (MF)” was significantly enriched, consistent with its role in condensing genomic DNA during the persistence phase of *Mycobacterium tuberculosis* (Matsumoto et al., 1999). In the case of Rho, “mRNA binding (MF)” was enriched, supporting the idea that Rho may drive phase separation by sensing cellular nutrient conditions through mRNA interactions (Krypotou et al., 2023). For SPT16, “regulation of transcription by RNA polymerase II (MF)” was enriched, reflecting its role as part of the FACT chromatin remodeler complex (Mayanagi et al., 2019; Formosa and Winston, 2020). The term “heparin binding (MF)” was enriched for Aβ,* aligning with findings that heparin can either promote or inhibit Aβ peptide fibrillation (Zhou et al., 2022). Similarly, “mitotic spindle organization (BP)” was enriched for Tau, consistent with its known function in stabilizing microtubules (Connolly et al., 1977). Lastly, “membrane organization (BP)” was significantly enriched for VIPP1, supporting its essential role in forming thylakoid membranes in chloroplasts and cyanobacteria (Zhang et al., 2016) (Figure 2a).

Alignments of representative IDR sequences closely related to AR*, αSyn, or VIPP1 are shown in Figure 2b. These sequences did not exhibit global similarity to the query sequences. The highest observed sequence identity was 44% between AR* and PAXIP1, mainly due to a shared polyglutamine (polyQ) array. However, such mono-amino acid repeats were absent in the case of αSyn, where only short motifs like E-x (5)-P-x (2)-E or E-x-E were shared. In contrast, no clear consensus motifs were observed in the VIPP1 example.

These results demonstrate that IDRs with similar functional roles were clustered by the encoding vectors generated by IDRdecoder, albeit not in a clearly discrete manner. Importantly, proximity between encoding vectors did not necessarily indicate explicit sequence similarity or the presence of well-defined sequence motifs.

### 3.2 Prediction of interaction sites via transfer learning

In the second phase, IDRdecoder was trained to predict ligand interaction sites within IDRs. For the training dataset, relevant studies on IDR-targeted drug discovery were systematically reviewed under the following criteria: (1) The amino acid sequence of the target IDR was clearly defined. (2) Ligand-interacting residues were experimentally identified, for example*,* through chemical shift perturbation analysis. (3) The chemical identities of the potential drug molecules were explicitly characterized. This search yielded studies on Aβ (Scherzer-Attali et al., 2010), AR (Sadar, 2020), PTP1B (Krishnan et al., 2014), TipA (Habazettl et al., 2014), αSyn (Tatenhorst et al., 2016), cMyc (Follis et al., 2008), p27 (Iconaru et al., 2015), NUPR1 (Neira et al., 2017), and p53 (Ruan et al., 2020), providing a dataset of 9 IDR sequences encompassing 130 interacting sites and 11 potential drug compounds.

Initially, this dataset was intended to serve as training and validation data. However, due to its limited size, it was used solely as a validation dataset (referred to as DS-IDR-V). Consequently, the training dataset was constructed by mining the Protein Data Bank (PDB) for ligand-interacting peptide segments with a higher propensity for intrinsic disorder (Supplementary Figures S1b,c) (wwPDB Consortium, 2019). Existing methods leveraging PDB data primarily focused on interaction sites within Molecular Recognition Features (MoRFs) structured regions formed through interactions with native binding partners (Chen et al., 2022). In contrast, this study broadened the data scope beyond known MoRFs. The IDR tendencies of peptide segments from PDB proteins were assessed using IUPred2A (Meszaros et al., 2018), and sequences displaying higher disorder tendencies were selected. This process resulted in a training dataset, referred to as DS-PDB-T, comprising 57,692 sequences with 171,007 ligand-interacting sites (Supplementary Table S1).

In this training phase, only the 10,162,860 weights of the decoding component of IDRdecoder were retrained. The input matrices were generated using the same method as in the initial phase; however, the decoding (target) matrices were specifically constructed to represent only the ligand-interacting sites. Training was intentionally halted at epoch 260 to prevent overfitting, as the output matrices became excessively sparse and often empty beyond this point, with clear signs of overfitting emerging (Figure 1c).

**FIGURE 3:**
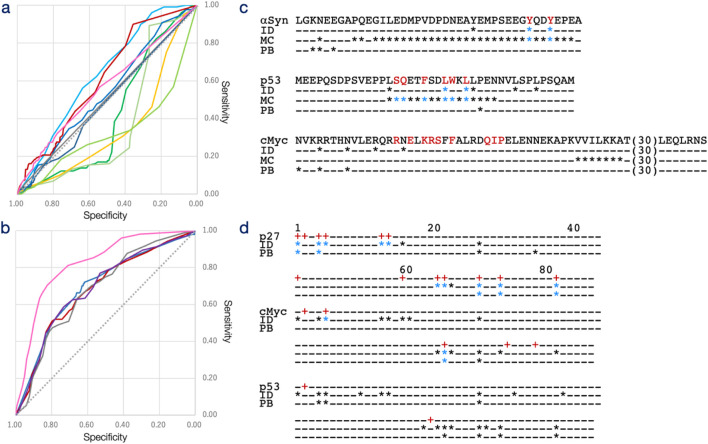
Receiver operating characteristic (ROC) curve of interacting site and interacting protogroup predictions. **(a)** Prediction results for interacting sites (DS-IDR-V) across varying thresholds are plotted with specificity on the horizontal axis and sensitivity on the vertical axis for IDRdecoder (red), IUPred2A (green), MoRF_CHiBi_ (sky blue), DeepDISOBind (DNA: yellow, RNA: yellow-green, and Protein: light green), and PrtoeinBERT (blue). IDRdecoder results for DS-PDB-V (magenta) and DS-PDB-N (gray) are also included. **(b)** Prediction results for interacting protogroups (DS-IDR-V) across varying thresholds are plotted with specificity on the horizontal axis and sensitivity on the vertical axis for IDRdecoder (red) and ProteinBERT (blue). Additionally, IDRdecoder results for DS-PDB-V (magenta), DS-PDB-N (gray), and the fully trained version (purple) are shown. **(c)** Examples of interacting site predictions at the optimal threshold are provided for αSyn (best case with a -log_10_P value of 3.77 for the chi-square test, top), p53 (intermediate case, 0.34, middle), and cMyc (worst case, 0.00, bottom; 30 amino acids of cMyc with no true or positive sites are omitted). **(d)** Examples of protogroup predictions (from 1 to 87) at the optimal threshold are shown for p27 - SJ572710 (best case, 7.05, top), cMyc-10058-F4 (intermediate case, 0.14, middle), and p53 – PKUMDL-RH-1047 (worst case, 0.04, bottom). True sites and protogroups (marked with a plus) are displayed in red on the top lines. Positive cases are indicated by an asterisk for IDRdecoder (ID), MoRFCHiBi (MC), and ProteinBERT (PB), while true-positive cases are shown in blue.

The decoding matrices produced predictions for ligand interaction sites, which were evaluated using the validation dataset DS-IDR-V (Figure 3a; Supplementary Table S3). The model’s performance was assessed through the area under the curve (AUC) of the receiver operating characteristic (ROC) curve across varying thresholds, revealing a moderate prediction capability with an AUC of 0.616. This performance was compared to several established methods, namely, IUPred2A, MoRF_CHiBi_ (Malhis and Gsponer, 2015), DeepDISOBind (Zhang et al., 2022), and ProteinBERT (retrained with DS-PDB-T) (Brandes et al., 2022), using the same validation set. However, this comparison was considered tentative since these existing methods are primarily designed to predict MoRFs involved in macromolecular interactions, rather than ligand-binding sites for small molecules.

Based on the AUC values, MoRFCHiBi demonstrated the highest predictive capability with an AUC of 0.637, followed by IDRdecoder with an AUC of 0.616 and ProteinBERT with an AUC of 0.538 (Table 1). The underlying models for these predictors differ significantly. MoRF_CHiBi_ integrates predictions based on sequence similarity, disorder propensity, and sequence conservation using Bayesian inference by referencing experimentally validated MoRFs. In contrast, ProteinBERT is a BERT (bidirectional encoder representations from transformers)-based model pretrained on over 10^8^ million protein sequences and fine-tuned for site prediction purpose using the DS-PDB-T dataset. The performance of MoRF_CHiBi_ would suggest the information of evolutionary sequence conservation was beneficial for prediction of ligand-binding sites, although structures of drug molecules were in general different from the native binding partners. The ROC profiles highlighted a notable distinction between IDRdecoder and the other models—IDRdecoder exhibited superior performance in regions of higher specificity. The specificities at the optimal thresholds, determined by the positive likelihood ratio (PLR), were 0.954 for IDRdecoder, 0.804 for MoRFCHiBi, and 0.882 for ProteinBERT. These results demonstrated that IDRdecoder detect lower number of the interaction sites more accurately compared to the other methods. This feature of IDRdecoder might be preferable as site predictor in experimental drug design, which typically starts with few target sites and lead molecule, then refine the structures of drug by adding binding sites and modifying molecular structure of drugs.

**TABLE 1:** Prediction statistics at best threshold value.

Target	Model	Data set	Thr	TP	FP	FN	TN	Sen/TPR	Spe	Pre	FPR	Acc	F	AUC	-log_10_P	PLR
Site	IUPred2A	DS-IDR-V	0.42	100	406	30	154	0.769	0.275	0.198	0.725	0.368	0.314	0.416	0.445	1.061
	MoRF_CHiBi_		0.86	40	110	90	450	0.308	0.804	0.267	0.196	0.710	0.286	0.637	2.098	1.566
	DeepDISOBind-PRT		0.08	116	410	14	150	0.892	0.268	0.221	0.732	0.386	0.354	0.379	3.753	1.219
	DeepDISOBind-DNA		0.60	1	4	129	556	0.008	0.993	0.200	0.007	0.807	0.015	0.349	0.000	1.077
	DeepDISOBind-RNA		0.04	130	560	0	0	1.000	0.002	0.188	0.998	0.190	0.317	0.331	0.000	1.002
	ProteinBERT		0.10	20	66	110	494	0.154	0.882	0.233	0.118	0.745	0.185	0.538	0.480	1.305
	IDRdecoder		0.70	17	26	113	534	0.131	0.954	0.395	0.046	0.799	0.197	0.616	3.146	2.817
		DS-PDB-V	0.80	42	211	217	2080	0.162	0.908	0.166	0.092	0.832	0.164	0.563	3.278	1.761
		DS-PDB-N	0.80	602	15,569	17,458	610,360	0.033	0.975	0.037	0.025	0.949	0.035	0.507	12.030	1.340
PG	ProteinBERT	DS-IDR-V	0.05	69	188	35	357	0.663	0.655	0.268	0.345	0.656	0.382	0.694	8.643	1.923
	IDRdecoder		0.55	54	140	50	405	0.519	0.743	0.278	0.257	0.707	0.362	0.702	6.792	2.021
		DS-PDB-V	0.80	263	201	602	3,064	0.304	0.938	0.567	0.062	0.806	0.396	0.822	15.658	4.939
		DS-PDB-N	0.60	22,387	40,796	31,545	200,272	0.415	0.831	0.354	0.169	0.7548	0.382	0.680	15.658	2.453
	IDRdecoder-retrain	DS-IDR-V	0.75	46	91	58	454	0.442	0.833	0.336	0.167	0.770	0.382	0.688	9.178	2.649

The highest -log_10_(P-value) of the chi-square test of the confusion matrix (TP, FP, FN, TN) for each model-target pair.

Thr, threshold; TP, umber of true positive; FP, false positive; FN, false negative; TN, true negative; Sen, sensitivity; TPR, true positive rate; Spe, specificity; FPR, false positive rate; Acc, accuracy; F, F-measure; AUC, area under curve; PLR, positive likelihood ratio.

Examples illustrating the best, intermediate, and worst prediction outcomes for individual IDRs are shown in Figure 3c. For the IDR of αSyn, which interacts with the potential drug Fasudil (Supplementary Figure S2) via two Tyr residues (highlighted in red in Figure 3c), IDRdecoder successfully assigned higher scores to these Tyr residues compared to other amino acids. MoRF_CHiBi_ also correctly predicted these residues but tended to overpredict, identifying more positives than necessary, whereas ProteinBERT performed poorly in this case. In the intermediate case of p53, IDRdecoder highlighted Leu residues, correctly identifying two interacting sites, while MoRF_CHiBi_ accurately detected the entire array of interacting sites. In the worst case involving cMyc, IDRdecoder suggested some Arg and Asn residues, but none of the methods, including IDRdecoder, successfully identified the true interacting residues. These examples suggest that IDRdecoder tends to prioritize certain amino acids over others, depending on the input sequence, leading to varied prediction performance.

The performance of IDRdecoder was further validated using the evidenced IDR dataset (DS-PDB-V), which included peptide segments from the PDB with structural evidence of order-disorder transitions upon ligand interaction. This dataset consisted of 70 sequences with 259 interacting sites (Supplementary Table S1). IDRdecoder performance for DS-PDB-V was limited by showing an AUC of 0.563. Nevertheless, it still demonstrated a higher specificity of 0.908 at the best threshold (Table 1).

Since the training data were obtained from structured proteins in the PDB, there was concern that IDRdecoder might be biased toward structured regions rather than IDRs. To address this, the dataset DS-PDB-N was created similarly to DS-PDB-T but included peptide segments with lower IDR tendencies ranging from 0.0 to 0.3. The AUC for this negative dataset was relatively low (0.507), supporting the idea that IDRdecoder is somewhat specialized in predicting IDR interaction sites.

### 3.3 Prediction of interacting ligands

In the third step, IDRdecoder was trained to predict interacting small molecules. A major challenge in this phase was the vast number of potential small molecules compared to the limited training data. To overcome this, IDRdecoder was designed to predict a manageable subset of commonly observed atom groups called protogroups. Protogroups are small chemical compounds identified in the PDB that act as protein ligands and often appear as substructures in larger ligands (see Supplementary Figure S1e; Supplementary Table S5). A total of 87 of the most frequent protogroups were selected, and prediction vectors were prepared as one-hot vectors, where only the protogroup interacting with each input sequence in DS-PDB-T was set to 1. During this step, the encoding and decoding parts were fixed, and 7,750,573 weights in the predicting part were trained. Training was completed at epoch 700.

The prediction capability of IDRdecoder was evaluated using the datasets DS-IDR-V and DS-PDB-V (Figure 3b; Supplementary Table S4). To date, no established methods exist for predicting small molecules interacting with IDRs. Therefore, IDRdecoder’s performance was compared to ProteinBERT, which had been fine-tuned on DS-PDB-T for classification tasks. The AUCs for IDRdecoder and ProteinBERT on DS-IDR-V were 0.702 and 0.694, respectively, while IDRdecoder achieved an AUC of 0.822 on DS-PDB-V (Table 1). Overall, IDRdecoder’s performance was comparable to ProteinBERT’s (Figure 2b).

Examples of predictions with the best, intermediate, and worst outcomes for individual IDRs are shown in Figure 3d. In the best-case scenario with p27, IDRdecoder correctly identified 10 out of 13 true protogroups of the potential drug SJ572710 (Supplementary Figure S2), with only three false positives. In contrast, for the intermediate (cMyc with 10058-F4) and worst (p53 with PKUMDL-RH-1047) cases, both IDRdecoder and ProeinBERT failed to detect more than half of the true protogroups. Notably, IDRdecoder consistently underestimated protogroup 2 (benzene), a commonly used and crucial atom group in many potential drugs targeting IDRs (Supplementary Figure S1).

**FIGURE 4:**
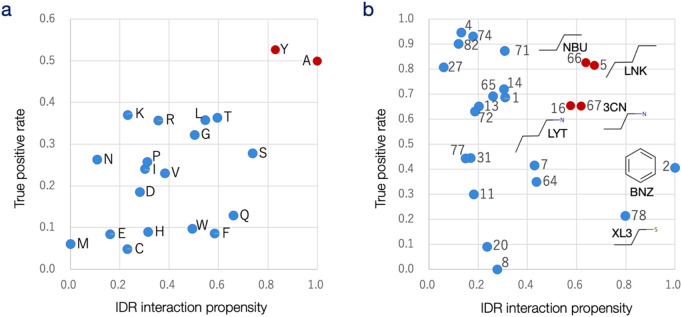
Analysis of prediction results for each amino acid and protogroup. **(a)** The true-positive rate (TPR; vertical axis) for each amino acid in the DS-IDR-V and DS-PDB-V datasets is plotted against the IDR propensity of each amino acid for interacting sites (horizontal axis). The correlation coefficient between amino acid propensity and TPR is 0.57, indicating a moderate positive correlation. **(b)** The true-positive rate (vertical axis) for each protogroup in the DS-IDR-V and DS-PDB-V datasets is plotted against the protogroup’s propensity for binding to IDR (horizontal axis). Protogroup numbers are labeled, and the chemical formulas along with the corresponding PDB ligand codes are provided for six protogroups with the highest propensities. The correlation coefficient between protogroup propensity and TPR is −0.25, suggesting a weak negative correlation.

IDRdecoder’s AUC for the negative dataset DS-PDB-N was 0.680, comparable to its performance on DS-IDR-V. This result suggests that, unlike in interaction site prediction, IDRdecoder may be more tuned to general protein ligands rather than being specifically optimized for IDR-targeted ligands.

Finally, the entire 25,602,653 weights in IDRdecoder were retrained simultaneously using the DS-PDB-T dataset for comparative analysis (Supplementary Figure S1f). This approach was taken because the stepwise transfer learning process may have limited the performance of IDRdecoder’s downstream components, particularly the protogroup prediction module. In this retraining, the initial model and training conditions were consistent with those used in the third step, except that all weight constraints were removed. As a result, the learning process displayed rapid signs of overfitting, leading to early termination at epoch 50. The fully trained model achieved an AUC of 0.688, showing no significant improvement over the stepwise trained model, which had an AUC of 0.683 (Table 1).

## 4 Discussion

IDRdecoder was designed to predict ligand interaction sites and ligand types for IDRs through a stepwise process. In the first step, sequence features were extracted as encoding vectors using an autoencoder. The PC map of these encoding vectors revealed that IDRdecoder had a limited ability to cluster IDRs. This limitation likely stems from the inherently low-complexity sequences of IDRs and their high variability, even among homologous proteins (Figure 2a). Despite this, results from GO enrichment analyses indicated that the feature extraction process was effective. GO terms uniquely associated with well-characterized IDRs, such as AR and αSyn, were significantly enriched near their respective IDRs.

This analysis did not rely on detecting homology, as homologous proteins identified through similarity searches were excluded. Instead, IDRdecoder appeared to recognize simple motifs, typically composed of two amino acids (Figure 2b). This is reasonable given that the input matrix is a three-dimensional representation of the sequence, capturing the relative frequency and positional separation of amino acid pairs. Consequently, two-residue motifs characteristic of IDRs were likely encoded in the encoding vectors, and the observed GO enrichment patterns suggest that some of these motifs contribute to specific biological functions (Supplementary Figure S4).

It is important to note that not all enriched GO terms directly reflected the functional roles of IDRs, and some were potentially misleading for functional predictions. For instance, the term “ATP-dependent chromatin remodeler activity (MF)” was significantly enriched for SPT16, despite SPT16 being a component of an ATP-independent chromatin remodeler (Supplementary Table S2) (Valieva et al., 2016). Similarly, “positive regulation of transcription by RNA polymerase II” and “positive regulation of transcription by RNA polymerase I″ were suggested for AR, though the latter is not functionally accurate for this protein (Panigrahi and O'Malley, 2021). Additionally, the IDRs selected for GO enrichment analysis were mainly clustered near the densely populated center of the distribution space (Figure 2a), leaving uncertainty about whether the observed functional clustering holds consistently across the entire distribution.

In the second step, IDRdecoder focused on predicting small molecule interaction sites within IDRs—a critical aspect of this study. Existing prediction methods are primarily designed for interactions with macromolecules such as proteins or DNA, and, more notably, no validated dataset previously existed for training small molecule interaction predictions in IDRs. To address this, the training dataset (DS-PDB-T) was constructed by integrating molecular interaction data from known non-disordered peptide segments that displayed a relatively higher propensity for intrinsic disorder. As a result, IDRdecoder achieved moderately better predictive performance compared to other methods when evaluated against the validation dataset (DS-IDR-V). Conversely, its performance was lower on the negative dataset (DS-PDB-N), which may further support the validity and specificity of this approach (Figure 3a).

However, closer examination of the prediction results revealed a potential bias in IDRdecoder’s predictions. Specifically, the individual prediction scores for certain IDRs, such as αSyn and TipA, were noticeably higher than for others. This aligns with experimental findings where Tyr residues were frequently identified as interaction sites for these proteins (Figure 3c; Supplementary Table S3). IDRdecoder appeared to favor specific amino acids depending on the input sequence, consistently assigning higher scores to Tyr for αSyn and TipA, Ala for AR and Aβ, Pro for PTPB1, and Leu for p53. This pattern suggests that IDRdecoder may not be adequately trained to interpret the broader context of amino acid sequences and to evaluate residue sites in a context-dependent manner. This limitation could stem from insufficient training data or inherent constraints in the model’s architecture.

Given that the input (20 × 20 × 20) matrix of IDRdecoder is a 3D representation of amino acid sequences—reflecting the relative frequency of amino acid pairs—this data representation may contribute to the observed bias toward specific amino acids. IDRdecoder employed the three-dimensional matrix input to accept sequences in different lengths, which was prominent difference from transformers (ProteinBERT), which use positional encoding for same purpose. The apparent disadvantage of the matrix presentation is that contexts of sequence, for which positional encoding can maintain, are largely lost in processing. It might explain the limitation of current model. On the other hand, the matrix can explicitly keep two-residue motif information (Supplementary Figure S4). It suggests that IDRdecoder can be further developed as an IDR-motif identifier or classifier by combining the two-residue motifs embedded in the encoding vectors. Since the performance of IDRdecoder and ProteinBETRT were comparable, superiority between the models would not be concluded in this case.

When the prediction performance for each amino acid, measured by the true-positive rate (TPR), was compared to the IDR propensity (the relative likelihood of each amino acid participating in IDR interactions in DS-IDR-V vs. in ordered proteins), a moderate correlation coefficient of 0.57 was observed (Figure 4a). This result suggests that amino acids like Ala and Tyr are favored for small molecule interaction sites within IDRs and that IDRdecoder effectively captured this trend by frequently prioritizing these residues. Interestingly, according to the previous studies, Tyr is ranked 3rd lowest in IDR-promoting propensity, next to other aromatic amino acids Trp and Phe (Campen et al., 2008), but is relatively preferred for interacting sites in ordered proteins (Soga et al., 2007). Probably, Tyr is relatively irregular in IDR sequence and thus appropriate for a target site.

In the third step, IDRdecoder predicts interacting molecular substructures. Due to limited training data, the prediction target was restricted to the 87 most frequent molecular substructures (protogroups) instead of entire molecules. To the best of our knowledge, no existing prediction methods have been designed for this specific purpose, and an extensive performance comparison was not feasible in this study, except with ProteinBERT. Consequently, IDRdecoder demonstrated significant prediction capability (Figure 3b). However, a concern arose from the results: the performance of protogroup prediction appeared independent of that for the interacting site. The protogroup prediction capability generally surpassed that of the interacting site, notably in DS-PDB-V, which showed AUCs of 0.822 for the former and 0.563 for the latter (Table 1). This could indicate a bias toward non-IDR interactions, likely due to the training data being sourced from the PDB, which was not ideal for the study’s objective.

The performance (TPR) for each protogroup was compared with the IDR propensity—the relative tendency of each protogroup to appear in IDR-interacting molecules compared to ligands of general ordered proteins (Figure 4b). Higher IDR interaction propensity was observed for protogroups 2 (benzene), 78 (propane-1-thiol), 5 (pentane), 66 (*N*-butane), 67 (3-aminopropane), and 16 (butylamine). As is common with general drug candidate molecules, most compounds in DS-IDR-V contain aromatic groups, particularly benzene (Supplementary Figure S2). Interestingly, however, the TPR for benzene (protogroup 2, the second most frequent substructure in general PDB ligands) did not rank higher in this analysis. This trend of downgrading benzene was also evident in individual prediction cases (Figure 3d).

The high IDR propensity of benzene might come from the fact that most of the molecules in the DS-IDR-V were repositioned drugs, in which cyclic structures were preferred to deal with entropy loss in binding in the original design process. The result might suggest that drug-likeness was relatively lower importance for ligands for IDRs. Aromatic rings are typically favored as substructures in protein ligands because they offer a larger interaction surface area without greatly reducing conformational entropy. Although this should also apply to IDR interactions, the prediction results suggest that molecular flexibility may be a crucial factor in IDR-drug design. The IDR propensities revealed a preference for alkyl groups, such as propane (protogroup 67), butane (16 and 66), and pentane (5), with higher prediction capability observed for these protogroups (Figure 4b). It was speculated that since IDRs lack a preformed structure, their interactions with ligand molecules likely occur through induced fitting. This process could be facilitated if the ligand molecules’ conformations also adjust inductively, provided the interactions compensate for entropy loss. This interpretation would require to be confirmed against larger data set in future.

As mentioned, the three steps of transfer learning were employed in this study to predict IDR classification, interaction sites, and interacting groups. Since this strategy could potentially reduce downstream performance, IDRdecoder was retrained without constraints for validation purposes (Figure 3b; Supplementary Figure S1f). However, this retraining quickly led to overfitting and did not show significant improvement, which may justify the use of the stepwise transfer-learning strategy for effectively suppressing overfitting.

In summary, IDRdecoder was proposed as a predictive tool for IDR-drug discovery by addressing the lack of training data. It demonstrated moderately improved performance compared to some existing methods. It is unlikely that the issue of limited training data will be resolved quickly. The analysis of prediction results revealed potential characteristics relevant to IDR-drug design. For instance, Tyr and Ala appear to be preferred target sites on IDRs, and alkyl groups are favored substructures in ligands. However, these conclusions should be considered tentative due to the limited size of the available dataset. Due to the data limitation, IDRdecoder was trained against the data from the known 3D structures, which remained a probability that the model was not fully trained against IDRs and biased toward structured regions of protein. Although IDRdecoder predict binding protogroups, it does not suggest how these protogroups should be arranged in a whole molecular structure of drug. The training for predicting protogroup combinations would require a significantly larger dataset. These are major limitations of the current IDRdecoder, which should be addressed in the future work.

## Data Availability

The datasets presented in this study can be found in online repositories. The names of the repository/repositories and accession number(s) can be found in the article/Supplementary Material.
