# Supplementary material for: IDRdecoder: a machine learning approach for rational drug discovery toward intrinsically disordered regions
Source: Front Bioinform. 2025 Jul 18;5:1627836. doi: 10.3389/fbinf.2025.1627836 (PMC12313641; doi:10.3389/fbinf.2025.1627836)
Supplement: Supplementary file 1 [file DataSheet1.pdf]

SUPPLEMANTAL INFORMATION

IDRdecoder: A machine learning approach for rational drug discovery toward intrinsically disordered regions

Clara Shionyu-Mitusyama(0009-0003-3186-2439),[1] Satoshi Ohmori,[1] Subaru Hirata,[2] Hirokazu Ishida(0000-0001-7474-2064),[1] Tsuyoshi Shirai(0000-0002-2506-5738)[1,2,*]

*Corresponding author: Tsuyoshi Shirai.

E-mail: t_shirai@nagahama-i-bio.ac.jp

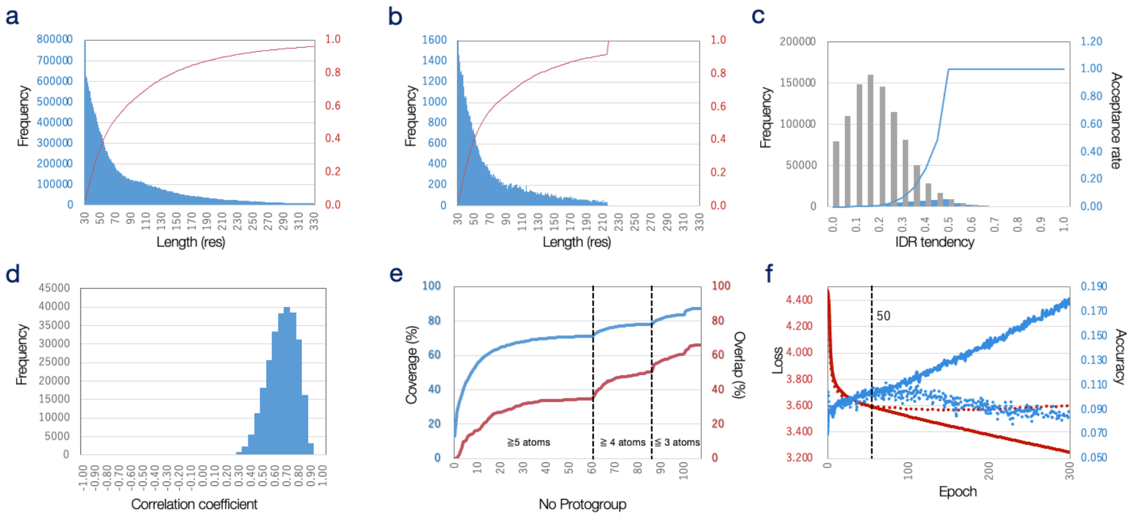

Figure S1. Statistics of IDRdecoder datasets and training. (a) The distribution of sequence lengths in the DS-IDR dataset is shown, with the frequency of sequences (vertical axis, scaled on the left) plotted against sequence length in residues (horizontal axis). The cumulative fraction of sequences is represented by the red curve, scaled on the right. (b) The distribution of sequence lengths for segments extracted from the PDB (DS-PDB) is illustrated. Sequence frequency (vertical axis, scaled on the left) is plotted against the number of residues (horizontal axis), with the cumulative fraction depicted by the red curve, scaled on the right. (c) The distribution of segments selected for the DS-PDB-T dataset, based on ligand interaction and IDR propensity from DS-PDB, is presented. The frequency of segments (vertical axis, scaled on the left) is plotted against IDR propensity (horizontal axis). Gray bars represent all segments interacting with ligands (including those selected), while blue bars indicate segments specifically chosen for DS-PDB-T. The blue curve, scaled on the right, shows the selection rate for each bin. (d) Distribution of correlation coefficients between input and decoding matrices of IDRdecoder. The vertical and horizontal axes are frequency and coefficient ranges, respectively. (e) The atom coverage of protogroups across all PDB ligands is shown, with the horizontal axis representing the protogroup number and the vertical axis indicating the cumulative fraction of atoms covered. The blue line shows the fraction of atoms covered, while the red line shows the overlap between protogroups. Protogroups with at least five heavy atoms were selected up to group 62, after which the threshold was reduced to four atoms up to group 87 and three atoms up to group 101. (f) The learning curve of IDRdecoder during unconstrained training is presented. The loss value (left vertical axis, in red) and accuracy metrics (right vertical axis, in blue) are plotted over 300 epochs. The dotted line marks the training termination at epoch 50.

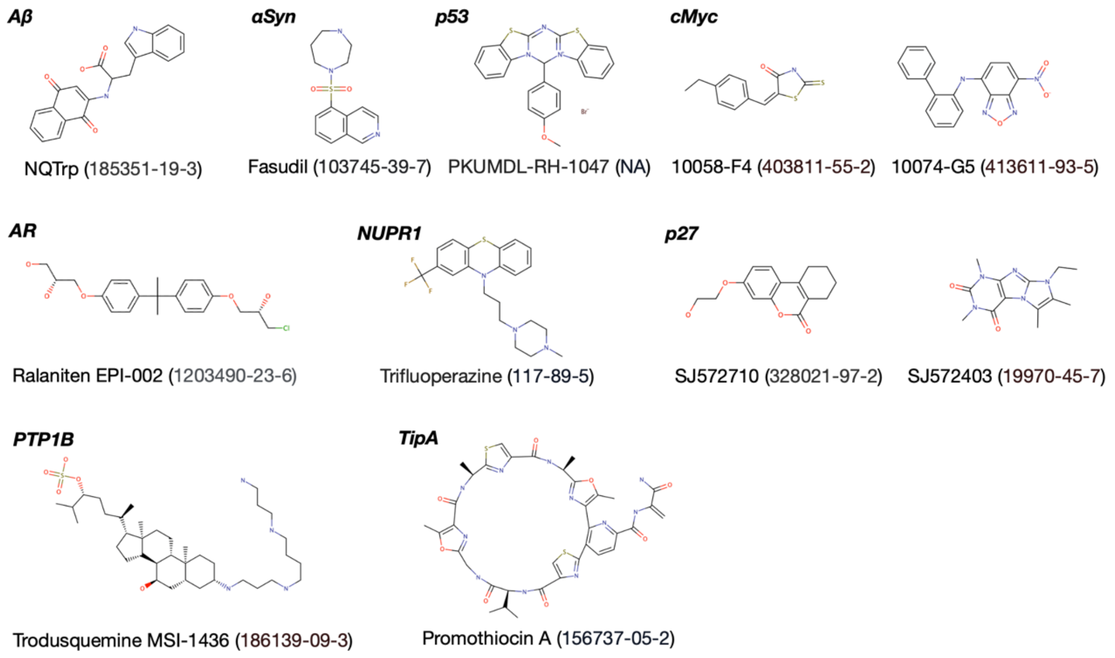

Figure S2. Chemical formula and names of potential drug molecules for IDRs in DS-IDR-V. In parentheses are CAS numbers.

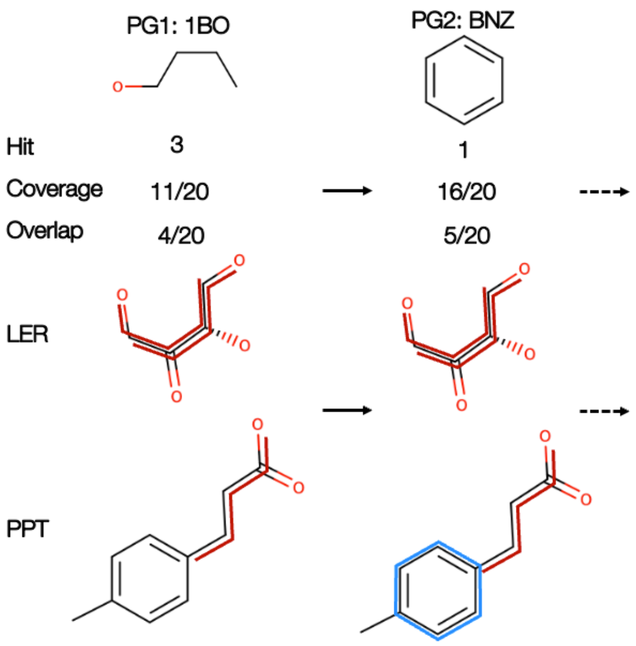

Figure S3. Scheme for protogroup identification. The protogroup identification process was schematically explained by showing only two PDB ligands (LER and PPT) and two protogroup PDB ligands (1BO and BNZ). The ligand molecules in PDB were compared and the frequency with which a small molecule matched to another was counted. In the presented case, 1BO matched twice and once for LER and PPT, respectively, and it covered 11/20 of all atoms with 4/20 overlap (fraction of atoms which matched to protogroups more than once). The most frequently matched molecule was selected as first protogroup. The atoms already matched to protogroup were excluded from further consideration. In the remaining groups, BNZ matched PPT, and selected second protogroup. The coverage and overlap became 16/20 and 5/20, respectively. The protogroups were identified by repeating these processes.

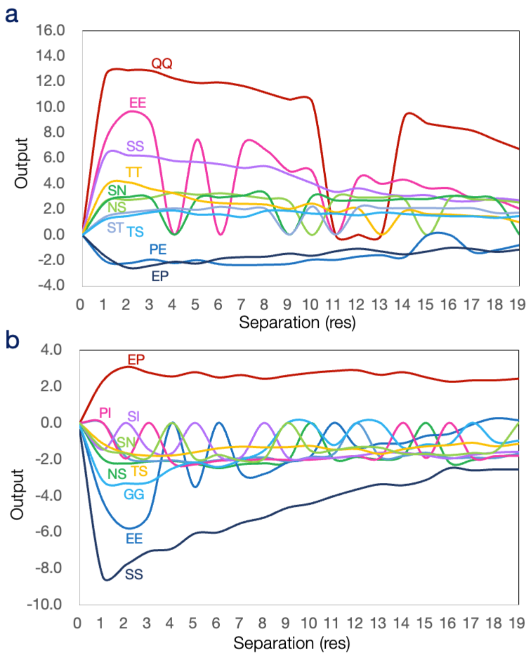

c

<table>
<tr><th rowspan="2">Non-zero element number l</th><th colspan="6">Dummy element value v</th></tr>
<tr><th>10</th><th>5</th><th>1</th><th>-1</th><th>-5</th><th>-10</th></tr>
<tr><td>1</td><td>G-x(14)-G</td><td>G-x(17)-G</td><td>Q-x-Q</td><td>E-x-P</td><td colspan="2">E-E</td></tr>
<tr><td>2</td><td colspan="2">T-T</td><td>S-S</td><td colspan="3">G-x(14)-G</td></tr>
<tr><td>3</td><td>S-x(9)-S</td><td>S-S</td><td colspan="3">T-T</td><td>T-x(6)-T</td></tr>
<tr><td>4</td><td>S-x(6)-S</td><td colspan="3">S-x(9)-S</td><td colspan="2">S-x(15)-S</td></tr>
<tr><td>5</td><td colspan="2">S-S</td><td colspan="2">S-x(6)-S</td><td colspan="2">S-x(9)-S</td></tr>
<tr><td>6</td><td>T-x(7)-T</td><td colspan="2">S-S</td><td colspan="3">S-S</td></tr>
<tr><td>7</td><td colspan="2">S-S</td><td colspan="4">T-x(7)-T</td></tr>
<tr><td>8</td><td colspan="6">S-S</td></tr>
<tr><td>9</td><td colspan="6">S-S</td></tr>
<tr><td>10</td><td colspan="2">S-x(18)-S</td><td colspan="4">S-S</td></tr>
<tr><td>11</td><td>S-S</td><td colspan="5">S-x(18)-S</td></tr>
<tr><td>12</td><td colspan="2">S-x(9)-E</td><td>T-x(5)-T</td><td colspan="3">S-S</td></tr>
<tr><td>13</td><td>T-x-S</td><td>T-x(5)-T</td><td colspan="4">S-x(9)-E</td></tr>
<tr><td>14</td><td colspan="4">T-x-S</td><td colspan="2">T-x(5)-T</td></tr>
<tr><td>15</td><td>N-N</td><td>S-x(3)-E</td><td colspan="2">T-x-S</td><td colspan="2">T-x(18)-T</td></tr>
<tr><td>16</td><td>T-x-E</td><td>S-x(3)-E</td><td colspan="2">N-N</td><td>N-x(18)-N</td><td>T-x(8)-T</td></tr>
<tr><td>17</td><td colspan="2">E-x(12)-T</td><td>T-x-E</td><td colspan="2">N-x(18)-N</td><td>T-x(14)-T</td></tr>
<tr><td>18</td><td>N-N</td><td>S-x(7)-S</td><td colspan="4">E-x(12)-T</td></tr>
<tr><td>19</td><td>E-x(11)-E</td><td>N-x(18)-N</td><td colspan="4">N-N</td></tr>
<tr><td>20</td><td colspan="2">P-x(5)-S</td><td colspan="2">E-x(11)-E</td><td colspan="2">S-x(4)-E</td></tr>
</table>

Figure S4. Embedding scheme of IDRdecoder. To analyze the embedding scheme of the encoding part of IDRdecoder, dummy encoding vectors $v_e(l)$ were input into the decoding part, and the resulting output values in the decoding matrices $f_d(a_i, a_j, k)$ were examined. In each dummy encoding vector, all elements were set to zero except for one selected element. For example $v_e(l = 3, v = -1) = \{0, 0, -1, 0, 0, 0, 0, 0, 0, 0, 0, 0, 0, 0, 0, 0, 0, 0, 0, 0\}$ when the *l* = 3 element was selected and set to *v* = -1. (a) An example output from $v_e(l = 1, v = 1)$ and (b) $v_e(l = 1, v = -1)$. The output values (vertical axis) in $f_d(a_i, a_j, k)$ for amino acid pairs $a_i a_j$ plotted against the separation residue number *k* (horizontal axis). Only selected amino acid pairs that produced higher $\sum |f_d(a_i, a_j, k)|$ values were plotted. These examples suggest that an element represents a specific pair of amino acids, such as $a_i a_j$ = QQ or EP in this case. The sign of the element may encode different amino acid pairs positive for QQ and negative for EP in this instance. It is speculated that these pairs rarely co-occur within the same sequence. (c) The amino acid pairs and separations that produced the highest output values for $v_e(l, v)$ with non-zero element *l* (ranging from 1 to 20 on the vertical axis) and input dummy element values *v* of 10, 5, 1, -1, −5, and -10 (on the horizontal axis) are shown. The results indicate that frequently encoded amino acids were S, T, N, and E, aligning with their general IDR propensity. The encoded amino acid pairs tended to be proximal, such as S-S (*l* = 5, 8, 9, or 11 and *v* = 10), N-N (*l* = 15 or 18 and *v* = 10), or T-x-S (*l* = 13, 14, or 15). An element may encode different pairs of amino acids depending on its magnitude. For example G-x(14)-G, G-x(17)-G, Q-x-Q, E-x-P, and E-E were observed for *l* = 1 with *v* = 10, 5, 1, -1, −5, and -10, respectively.

Table S1. Dataset statistics

Dataset	No. seq	No. res	<Length>	Max length	Min length	No. int. site[*1]	No. int. PG[*1]
DS-IDR[*2]	26,480,862	2,884,379,533	109	17,252	31	NA	NA
DS-IDR-V[*3]	9	652	72	145	39	145	184
DS-PDB-T[*4]	57,448	4,903,731	85	219	31	171,007	57,692
DS-PDB-V[*5]	70	2,550	36	67	26	259	1,302
DS-PDB-N[*6]	5,000	643,989	129	217	7	18,060	94,091

*1 No. int. site and No. int. are a summation of true interacting sites and protogroups for the IDRs in each data set, respectively.

*2 Predicted IDR sequences from RefSeq (GCF) genome assembly database for training. Similar sequences from different strains of same species were excluded.

*3 IDR sequences of experimentally evaluated for drug target for evaluation.

*4 Randomly selected PG interacting fragments of PDB sequences with relatively higher IDR tendency (> 0.5 IUPred2A score) for training.

*5 PG interacting fragments of PDB sequences with structural evidence of disorder in ligand unbound state for evaluation.

*6 Randomly selected PG interacting fragments of PDB sequences with relatively lower IDR tendency (< 0.5 IUPred2A score) for evaluation and comparison.

Table S2. GO enrichment analysis

AR*:NP_000035.2:1-168[*1]			αSyn:NP_000336.1:100-140			MDP1:WP_003415107.1:100-214			Rho:WP_003898814.1:1-216		
Term	Cat[*2]	Pv[*3]	Term	Cat	Pv	Term	Cat	Pv	Term	Cat	Pv
regulation of transcription by RNA polymerase II	BP	16.2	high voltage-gated calcium channel activity	MF	9.0	structural constituent of chromatin	MF	43.6	RNA binding	MF	21.0
transcription by RNA polymerase II	BP	14.8	calcium ion import across plasma membrane	BP	8.2	nucleosome assembly	BP	39.2	mRNA binding	MF	17.2
mediator complex	CC	12.0	voltage-gated calcium channel complex	CC	8.2	Nucleosome	MF	33.3	mRNA export from nucleus	BP	13.0
DNA-binding transcription factor activity	MF	10.9	voltage-gated calcium channel activity	MF	7.8	Chromatin	MF	22.6	RNA export from nucleus	BP	12.7
sequence-specific DNA binding	MF	10.2	calcium ion import	BP	7.5	DNA binding	MF	15.1	ubiquitin binding	MF	10.4
positive regulation of transcription by RNA polymerase II	BP	9.7	calcium channel activity	MF	6.9	negative regulation of DNA recombination	BP	12.9	nucleus	CC	10.3
positive regulation of transcription by RNA polymerase I	BP	9.6	plasma membrane	CC	5.6	chromosome condensation	BP	11.5	ubiquitin-dependent protein catabolic process	BP	9.1
DNA-binding transcription factor activity, RNA polymerase II-specific	MF	8.4	catenin complex	CC	5.5	chromosome	CC	11.4	proteasome-mediated ubiquitin-dependent protein catabolic process	BP	7.6
cis-regulatory region sequence-specific DNA binding	MF	8.1	transport along microtubule	BP	5.4	double-stranded DNA binding	MF	11.2	positive regulation of translation	BP	7.4
RNA polymerase II cis-regulatory region	MF	7.7	protein ubiquitination	BP	5.2	DNA recombination	BP	10.6	protein catabolic process	BP	6.9

sequence-specific DNA binding											
DNA binding	MF	7.5	membrane	MF	5.1	nucleosomal DNA binding	MF	10.0	regulation of dendrite morphogenesis	BP	6.7
protein dimerization activity	MF	7.5	dynein complex	CC	4.7	calcium-dependent cysteine-type endopeptidase inhibitor activity	MF	3.9	membrane fusion	BP	6.4
MLL3/4 complex	CC	5.5	cytoplasmic dynein complex	CC	4.6	inhibition of cysteine-type endopeptidase activity	BP	3.9	nuclear membrane reassembly	BP	6.4
transcription coactivator activity	MF	4.3	dynein heavy chain binding	MF	4.6	endopeptidase inhibitor activity	MF	2.4	dendrite morphogenesis	BP	6.3
nuclear receptor coactivator activity	MF	4.2	dynein light chain binding	MF	4.6	cysteine-type endopeptidase activity	MF	2.3	neuronal ribonucleoprotein granule	CC	6.2
anterior/posterior pattern specification	BP	3.9	RNA-DNA hybrid ribonuclease activity	MF	4.5	euchromatin	CC	2.1	regulation of dendritic spine development	BP	6.2
transcription regulator complex	CC	3.7	DNA replication-dependent chromatin assembly	BP	4.3	mitochondrial chromosome	CC	2.1	regulation of modification of synaptic structure	BP	6.2
negative regulation of TOR signaling	BP	3.2	beta-catenin binding	MF	4.1	mitochondrial genome maintenance	BP	2.1	regulation of translation at postsynapse, modulating synaptic transmission	BP	6.2
neurotrophin TRK receptor signaling pathway	BP	3.2	beta-catenin destruction complex	CC	4.1	mitochondrial nucleoid	CC	2.1	translation regulator activity	MF	6.2
response to hormone	BP	3.2	gamma-catenin binding	MF	4.1	recombinational repair	BP	2.1	autophagosome assembly	BP	6.1

SPT16:NP_009123.1:926-1047			Ab*:NP_000475.1:226-284			Tau:NP_001116538.2:1-601			VIPP1:NP_001322349.1:221-259		
Term	Cat	Pv	Term	Cat	Pv	Term	Cat	Pv	Term	Cat	Pv
ATP-dependent chromatin remodeler activity	MF	14.2	endoplasmic reticulum calcium ion homeostasis	BP	11.3	protein phosphorylation	BP	33.2	plasma membrane organization	BP	4.6
regulation of cell shape	BP	9.5	serine-type endopeptidase inhibitor activity	MF	6.8	kinase activity	MF	25.1	membrane organization	BP	4.3
protein binding	MF	9.5	transition metal ion binding	MF	6.8	cardiac muscle cell development	BP	19.8	response to oxidative stress	BP	4.3
SWI/SNF complex	CC	8.2	heparin binding	MF	6.4	cell development	BP	19.1	centrosome	CC	4.2
nucleolus	CC	7.9	termination of RNA polymerase I transcription	BP	6.4	protein kinase activity	MF	18.6	cytoplasmic side of plasma membrane	CC	3.6
photoreceptor connecting cilium	BP	6.4	rRNA primary transcript binding	MF	6.1	ATP binding	MF	15.3	mitotic cell cycle	BP	3.3
phosphatase regulator activity	MF	6.0	transcription initiation at RNA polymerase I promoter	BP	6.1	cerebral cortex development	BP	14.1	myosin complex	CC	3.3
regulation of transcription by RNA polymerase II	BP	5.8	endoplasmic reticulum	CC	6.0	cell population proliferation	BP	12.5	adherens junction maintenance	BP	3.2
chromatin	MF	5.7	endopeptidase inhibitor activity	MF	5.8	mitotic spindle organization	BP	10.7	cyclin-dependent protein serine/threonine kinase activity	BP	3.2
transcription by RNA polymerase II	BP	5.4	caveola	CC	5.3	protein serine/threonine kinase activity	BP	9.4	peroxisome proliferator activated receptor binding	MF	3.2
cilium organization	BP	5.2	nucleic acid binding	MF	5.0	actin filament	BP	9.4	PR-DUB complex	CC	2.8
nuclear receptor binding	MF	5.2	ATP hydrolysis activity	MF	4.6	myelination	BP	8.3	animal organ morphogenesis	BP	2.8

precatalytic spliceosome	CC	5.2	axonogenesis	BP	4.2	mitotic spindle	BP	7.8	antiporter activity	MF	2.8
3',5'-cyclic-AMP phosphodiesterase activity	MF	4.9	negative regulation of organ growth	BP	4.0	regulation of apoptotic process	BP	7.3	centriole replication	BP	2.8
A band	CC	4.9	regulation of hippo signaling	BP	4.0	apoptotic process	BP	6.8	regulation of mitotic cell cycle	BP	2.8
enzyme inhibitor activity	MF	4.9	maturation of SSU-rRNA from tricistronic rRNA transcript (SSU-rRNA, 5.8S rRNA, LSU-rRNA)	BP	3.7	transcription corepressor activity	MF	6.4	cyclin-dependent protein serine/threonine kinase inhibitor activity	MF	2.8
brahma complex	CC	4.7	hippo signaling	BP	3.7	spindle	CC	6.4	pre-mRNA 3'-splice site binding	MF	2.8
cysteine-type peptidase activity	MF	4.6	molecular adaptor activity	MF	3.7	actin monomer binding	MF	6.1	peroxisome	CC	2.7
RNA binding	MF	4.5	microtubule motor activity	MF	3.6	axonal growth cone	CC	6.1	transport	MF	2.7
U3 snoRNA binding	MF	4.4	ATP-dependent protein folding chaperone	MF	3.5	dendritic growth cone	CC	6.1	microfilament motor activity	MF	2.6

*1 Name of IDR is shown as protein name:Refseq ID:residue range.

*2 Category of GO term: Molecular Function (MF), Cellular Component (CC), Biological Process (BP).

*3 -$\log_{10}$P-value of binominal test.

Table S3. Interaction site prediction statistics[*1]

IDR[*2]	Interacting site[*3]	TP	FP	FN	TN	Acc	Sen	Spe	F	-log10P
αSyn:NP_000336.1:100-140	LGKNEEGAPQEGILEDMPVDPDNEAYEMPSEEGYQDYEPEA<br>---------------------------------*--*----<br>-------------------------*-------*--*----	2	1	0	38	0.975	0.995	0.974	0.798	3.774
AR:NP_000035.2:343-448	STLSLYKSGALDEAAAYQSRDYYNFPLALAGPPPPPPPPHPHARIKLENPLDYGS<br>*******************************-------------------*****<br>--------------*------------*-*-------------------------<br><br>AWAAAAAQCRYGDLASLHGAGAAGPGSGSPSAAASSSWHTLFTAEEGQLYG<br>*******************-----------*********************<br>---***--------------------------*------------------	7	0	69	30	0.349	0.092	1.000	0.169	0.698
cMyc:NP_002458.2:353-437	NVKRRTHNVLERQRRNELKRSFFALRDQIPELENNEKAPKVVILKKATAYILSVQ<br>--------------*-*-***-*----***-------------------------<br>---*---*---*---*---------------------------------------<br><br>AEEQKLISEEDLLRKRREQLKHKLEQLRNS<br>------------------------------<br>------------------------------	0	4	9	72	0.847	0.001	0.947	0.002	0.000
NUPR1:NP_001035948.1:1-100	MATFPPATSAPQQPPGPEDEDSSLDESDLYSLAHSYLGPLIMPMPTSPLTPALVT<br>--------**------------------*--**----------------------<br>--*<br><br>GGGGRKGRTKREAAANTNRPSPGGHERKLVTKLQNSERKKRGARR<br>*-----------------------*-----*--------------<br>-*---	0	2	8	90	0.900	0.001	0.978	0.002	0.000
P27:NP_004055.1:51-91	DMEEASQRKWNFDFQNHKPLEGKYEWQEVEKGSLPEFYYRPPRPPKGACKVPAQE<br>---------*-*-*---------*-*----------***----------------<br>------------------*----*----------*--**-**-**------*---	3	7	5	40	0.782	0.375	0.851	0.334	0.525
TipA:P0A4T9.1:1-150	PAADPRAHLRRQHELLSARIGKLQKMAAAVEQAMEARSMGINLTPEEKFEVFGDF<br>--*<br>---<br><br>DPDQYEEEVRERWGNTDAYRQSKE<br>----*---*---*-----*-----<br>----*-------------*-----	2	0	3	74	0.962	0.400	1.000	0.571	4.269

PTPB1:NP_002818.1:291-435	WKELSHEDLEPPPEHIPPPPRPPKRILEPHNGKCREFFPNHQWVKEETQEDKDCP<br>--------*-----**---------------------------*-----------<br>--*---*----------**---*---------------*----------------<br><br>IKEEKGSPLNAAPYGIESMSQDTEVRSRVVGGSLRGAQAASPAKGEPSLPEKDED<br>-------------------------*-*-*-----------------*-------<br>---<br><br>HALSYWKPFLVNMCVATVLTAGAYLCYRFLFNSNT<br>-----------------------------------<br>-----------------------------------	0	6	8	131	0.903	0.001	0.956	0.001	0.000
Ab:NP_000475.1:672-710	DAEFRHDSGYEVHHQKLVFFAEDVGSNKGAIIGLMVGGV<br>------------***--*****-----------------<br>-*------------------*--------*---------	1	2	7	29	0.769	0.126	0.935	0.183	0.000
p53:NP_000537.3:1-39	MEEPQSDPSVEPPLSQETFSDLWKLLPENNVLSPLPSQAM<br>--------------**--*--**-*---------------<br>-------------*-------*--**-----*--*-----	2	4	4	30	0.800	0.334	0.882	0.334	0.343

*1 Abbreviations: TP; umber of true positive, FP; false positive, FN; false negative, TN; true negative, Sen; sensitivity, Spe; specificity, Acc; accuracy.
*2 Name of IDR is shown as protein name:Refseq ID:residue range.

*3 Top line; amino acid sequence of IDR, middle line; true sites are indicated as asterisk, bottom line; sites predicted above threshold are indicated as asterisk.

Table S4. Interaction site prediction statistics for target-drug pairs[*1]

Target:Drug[*2]	Interacting PG[*3]	TP	FP	FN	TN	Acc	Sen	Spe	F	-log10P
αSyn:Fasudil	_*__*__________*__*________*________________<br>*__**_______**_*__________*_________________<br><br>____________________**__*_______*_________<br>____________________***___**_*_______*_____	4	10	5	40	0.746	0.445	0.800	0.348	0.611
AR:Ralaniten EPI-002	**_**_______**____________*_________________<br>___**__________**_________*_________________<br><br>_______________*____**____*__*_______*_____<br>_______________________*_____*_______*_____	5	3	8	43	0.813	0.385	0.935	0.476	1.920
cMyc:10058-F4	_*__*______________________________________<br>*__**_______**_**_________*_________________<br><br>____________________*________*___*________<br>____________________***___*__*_______*_____	2	12	3	42	0.746	0.400	0.778	0.211	0.138
cMyc:10074-G5	_*__*__________*__*_________________________<br>*__**_______**_**_________*_________________<br><br>____________________**____________*_______<br>____________________***___*__*_______*_____	4	10	3	42	0.779	0.571	0.808	0.381	1.088
NUPR1:Trifluoperazine	_*__*__*_______*___________*________________<br>*__**____*__**____________*____*______*_____<br><br>_________*_________*_**_**____*__*_________<br>__________________*_**____*__*_______*_____	2	13	11	33	0.593	0.154	0.717	0.143	0.249
P27:SJ572710	**_**_______**_____________________________<br>*__**_______**_*__________*_________________<br><br>*______________*____**____*__*_______*_____<br>____________________***___*__*_______*_____	10	3	2	44	0.915	0.833	0.936	0.800	7.049
P27:SJ572403	**_**_______**_____________________________<br>*__**_______**_*__________*_________________<br><br>*______________*____**____*__*_______*_____<br>____________________***___*__*_______*_____	2	11	4	42	0.746	0.334	0.792	0.211	0.071
TipA:Promothiocin A	____*_*___*____*__*__*________*_____________<br>*__**_______**_*__________*_________________	7	7	9	36	0.729	0.438	0.837	0.467	1.203

	---------*----------***--***--*-**---------<br>--------------------***---**-*-------*-----									
PTPB1:Trodusquemine	*---*--------*-*-----------*-----------------<br>*--**-------**-*----------*------------------<br><br>*--------------*-----**---*--*-------------<br>--------------------**----*--*-------*-----	7	5	3	44	0.864	0.700	0.898	0.636	3.925
Ab:NQTrp	**--*-*--------*--**------------------------<br>*--**-------**-**---------*-----------------<br><br>----------------------**---**---------------<br>--------------------***---**-*-------*-----	7	8	4	40	0.796	0.636	0.833	0.538	2.349
p53:PKUMDL-RH-1047	-*---<br>*--**----*--**-----------*----*------*-----<br><br>-------------------*-----------------------<br>------------------*-***---**-*-------*-----	0	17	2	40	0.678	0.005	0.702	0.001	0.041

*1 Abbreviations: TP; umber of true positive, FP; false positive, FN; false negative, TN; true negative, Sen; sensitivity, Spe; specificity, Acc; accuracy.

*2 Protein name and drug name are shown.

*3 Top line; true protogroups are indicated as asterisk, bottom line; protogroups predicted above threshold are indicated as asterisk

Table S5. Protogroups

No.	No. atoms	Code[*1]	Name	SMILES
1	5	1BO	1-Butanol	CCCCO
2	6	BNZ	Benzene	c1ccccc1
3	5	NML	N-Methylacetamide	CC(=O)NC
4	5	PDO	1,3-Propandiol	C(CO)CO
5	5	LNK	Pentane	CCCCC
6	6	P1R	Pyrimidine	c1cncnc1
7	5	ALA	Alanine	C[C@@H](C(=O)O)N
8	5	TEA	Triethylammonium ion	CC[NH+](CC)CC
9	5	PO4	Phosphate ion	[O-]P(=O)([O-])[O-]
10	5	AKR	Acrylic acid	C=CC(=O)O
11	6	0PY	Pyridine	c1ccncc1
12	5	IMD	Imidazole	c1c[nH+]c[nH]1
13	5	MOE	Methoxy-ethoxyl	COCC[O-]
14	5	SBT	2-Butanol	CC[C@H](C)O
15	5	MGX	1-Methylguanidine	[H]/N=C(/N)\NC
16	5	LYT	Butylamine	CCCCN
17	6	ATJ	Ethyl hydrogen phosphonate	CCO[P@H](=O)O
18	5	DMF	Dimethylformamide	CN(C)C=O
19	5	IBN	2-Methylpropan-1-amine	CC(C)CN
20	6	5MP	5-Methylpyrrole	Cc1cc[nH]c1
21	5	PZO	Pyrazole	c1c[nH]nc1
22	5	GM1	Aminomethylamide	C(C(=O)N)N
23	5	NMU	N-Methylurea	CNC(=O)N

24	6	SAR	Sarcosine	CNCC(=O)O
25	5	LOM	Thiazole	c1=csc=n1
26	6	MAS	Dimethylsulfonic amide	CN(C)S(=O)(=O)O
27	5	PGO	S-1,2-Propanediol	C[C@@H](CO)O
28	5	13D	1,3-Diaminopropane	C(CN)CN
29	5	TBU	Tertiary-butyl alcohol	CC(C)(C)O
30	5	MPR	2-Mercapto-propion aldehyde	C(CS)C=O
31	5	ROP	Propionamide	CCC(=O)N
32	6	EEE	Ethyl acetate	CCOC(=O)C
33	6	PIV	Pivalic acid	CC(C)(C)C(=O)O
34	7	MEC	Ethyl-carbamic acid methyl ester	CCNC(=O)OC
35	5	SO4	Sulfate ion	[O-]S(=O)(=O)[O-]
36	6	MR3	1-Methyl-1h-pyrrole	Cn1cccc1
37	5	NTB	Tertiary-butylamine	CC(C)(C)N
38	5	GLM	1-Amino-propan-2-one	CC(=O)CN
39	5	GLV	Glyoxylic acid	C(=O)C(=O)O
40	5	PGL	Aminomethylenephosphinic acid	C(N)P(=O)(O)O
41	9	MDN	Methylenediphosphonic acid	C(P(=O)(O)O)P(=O)(O)O
42	6	ESA	Ethanesulfonic acid	CCS(=O)(=O)O
43	5	MCR	Sulfanylacetic acid	C(C(=O)O)S
44	5	HAE	Acetohydroxamic acid	CC(=O)NO
45	9	OTT	(2e,4e,6e)-octa-2,4,6-Trienoic acid	C/C=C/C=C/C=C/C(=O)O
46	6	ETF	Trifluoroethanol	C(C(F)(F)F)O
47	7	CCP	Butylphosphonate	CCCCP(=O)(O)O

48	5	TSZ	Hydrazinecarbothioamide	C(=S)(N)NN
49	7	IBU	2-Methylpropyl hydrogen carbonate	CC(C)COC(=O)O
50	7	IPG	N-Isopropyl glycine	CC(C)NCC(=O)O
51	6	ITU	Ethylisothiourea	CCSC(=N)N
52	5	TMA	Tetramethylammonium ion	C[N+](C)(C)C
53	5	VO4	Vanadate ion	[O-][V](=O)([O-])[O-]
54	5	ALT	Thioalanine	C[C@@H](C(=S)O)N
55	6	B2A	Alanine boronic acid	B([C@H](C)N)(O)O
56	5	WO3	Tri-tungsten(vi) oxide complex	[O-][W](=O)(=O)[O-]
57	7	POA	Phosphonoacetaldehyde	C(C=O)P(=O)(O)O
58	6	DBU	(2e)-2-Aminobut-2-enoic acid	C\C=C(\C(=O)O)/N
59	7	BVC	1-Thien-3-ylmethanamine	c1cscc1CN
60	6	PNH	Pentan-2-one	CCCC(=O)C
61	5	NIE	Nitroethane	CC[N+](=O)[O-]
62	4	RGI	Methylcarbamic acid	CNC(=O)O
63	4	ACT	Acetate ion	CNC(=O)O
64	4	KEN	N,n-Dimethylmethanamine	CN(C)C
65	4	2ME	Methoxyethane	CCOC
66	4	NBU	N-Butane	CCCC
67	4	3CN	3-Aminopropane	CCCN
68	4	PHS	Phosphonic acid	OP(=O)O
69	4	CFT	Trifluoromethane	C(F)(F)F
70	4	AEM	2-Aminoethanimidic acid	[H]/N=C/CN
71	4	POL	N-Propanol	CCCO

72	4	ETA	Ethanolamine	C(CO)N
73	4	SO3	Sulfite ion	[O-]S(=O)[O-]
74	4	ACN	Acetone	CC(=O)C
75	4	TEE	2-Amino-ethenethiol	C(=CS)N
76	4	DMS	Dimethyl sulfoxide	CS(=O)C
77	4	ACM	Acetamide	CC(=O)N
78	4	XL3	Propane-1-thiol	CCCS
79	4	MTF	N-Methylthioformamide	CNC=S
80	4	NOE	Nitrosoethane	CCN=O
81	4	GB	Methylphosphonic acid ester group	CP(=O)(O)O
82	4	EDO	1,2-Ethanediol	C(CO)O
83	4	FES	Fe2/s2 (inorganic) cluster	S1[Fe]S[Fe]1
84	4	BME	b-Mercaptoethanol	C(CS)O
85	4	GAI	Guanidine	C(=N)(N)N
86	4	OUT	Carbamic acid	C(=O)(N)O
87	4	TOU	Thiourea	C(=S)(N)N

*1 PDB ligand code of each protogroup.

Supplementary description. Instruction and execution manual for IDRdecoder ver. 1.0

Prerequisites

Ensure Anaconda or Miniconda is installed on your system for managing environments.

Download and Install

Open a terminal (Command Prompt/PowerShell on Windows or Terminal on Linux/Mac). Clone the repository by running:

```
$ git clone https://github.com/emplics/idrdecoder.git
```

Navigate into the downloaded folder:

```
$ cd idrdecoder
```

Create and Activate the Conda Environment. Use the provided yaml file to create a Conda environment:

```
$ conda env create -f environment_{your platform}.yml
```

Activate the environment:

```
$ conda activate idrdecoder
```

Restore variable file:

```
for linux or mac:
$        cat        idr_aetrain_pg_240420.hd5/variables/x??        >
idr_aetrain_pg_240420.hd5/variables/variables.data-00000-of-00001
for windows:
$       copy        /B        idr_aetrain_pg_240420.hd5\variables\x??
idr_aetrain_pg_240420.hd5\variables\variables.data-00000-of-00001
```

Execution, input and output files

Execute IDRdecoder as follows: input file is multi-fasta sequences.

```
$ python idr_map_all_241214.py input-file(e.g. idr_target_sample.fas)
> output-file(e.g. idr_target_sample.dat)

 Argument: -h   : help
           -ist : threshold for interacting site (0.0-1.0, default
                  0.70)
```

```
          -pgt : threshold for protogroup (0.0-1.0, default 0.55)
```

Output file contains the following data:

```
Input: multi-fasta formatted amino acid sequences

Output:
 1) Standard output
  Column1 Column2  Column3-
  seq_n   name               : Sequence name of nth input
          seq                : Sequence
          ve                 : Encoding vector
          vepca              : Principle componets of encoding
                               vector
          issum              : Interaction site prediction summary
                               by ranking sites as 1-9a-z* (*=above
                               threshold)
          iscol    rnk       : Interaction site prediction rank
                   res       : Interaction site res no. and aa code
                   score     : Interaction site score
          islst              : Interaction site list
          pgsum              : Interacting protogroup prediction
                               summary by ranking protogroups as 1-
                               9a-z* (*=above thresold)
          pgcol    rnk       : Interacting protogroup prediction
                               rank
                   pg        : Interacting protogroup PDB code and
                                protogroup number
                   score     : Interacting protogroup score
                   name      : Interacting protogroup name
                   smiles    : Interacting protogroup smiles
          pglst              : Interacting protogroup list

 2) idr_map_landscape_01.png : landscape map of sequences with input
                                seq numbers
```
